# Meta-analysis of multiple microarray datasets reveals a common gene signature of metastasis in solid tumors

**DOI:** 10.1186/1755-8794-4-56

**Published:** 2011-07-07

**Authors:** Marla H Daves, Susan G Hilsenbeck, Ching C Lau, Tsz-Kwong Man

**Affiliations:** 1 Dan L. Duncan Cancer Center, Baylor College of Medicine, One Baylor Plaza, Houston, Texas, 77030, USA; 2 Texas Children's Cancer Center, Department of Pediatrics, Baylor College of Medicine, 6701 Fannin Street, Suite 1410, Houston, Texas, 77030, USA; 3 Breast Center, Baylor College of Medicine, One Baylor Plaza, Houston, Texas, 77030, USA

## Abstract

**Background:**

Metastasis is the number one cause of cancer deaths. Expression microarrays have been widely used to study metastasis in various types of cancer. We hypothesize that a meta-analysis of publicly available gene expression datasets in various tumor types can identify a signature of metastasis that is common to multiple tumor types. This common signature of metastasis may help us to understand the shared steps in the metastatic process and identify useful biomarkers that could predict metastatic risk.

**Methods:**

We identified 18 publicly available gene expression datasets in the Oncomine database comparing distant metastases to primary tumors in various solid tumors which met our eligibility criteria. We performed a meta-analysis using a modified permutation counting method in order to obtain a common gene signature of metastasis. We then validated this signature in independent datasets using gene set expression comparison analysis with the LS-statistic.

**Results:**

A common metastatic signature of 79 genes was identified in the metastatic lesions compared with primaries with a False Discovery Proportion of less than 0.1. Interestingly, all the genes in the signature, except one, were significantly down-regulated, suggesting that overcoming metastatic suppression may be a key feature common to all metastatic tumors. Pathway analysis of the significant genes showed that the genes were involved in known metastasis-associated pathways, such as integrin signaling, calcium signaling, and VEGF signaling. To validate the signature, we used an additional six expression datasets that were not used in the discovery study. Our results showed that the signature was significantly enriched in four validation sets with p-values less than 0.05.

**Conclusions:**

We have modified a previously published meta-analysis method and identified a common metastatic signature by comparing primary tumors versus metastases in various tumor types. This approach, as well as the gene signature identified, provides important insights to the common metastatic process and a foundation for future discoveries that could have broad application, such as drug discovery, metastasis prediction, and mechanistic studies.

## Background

Metastasis, the process involving the spread of cancer, accounts for greater than 90% of cancer deaths [1]. However, therapies to treat those patients with advanced disease are largely ineffective. It is, therefore, imperative that we improve the understanding of the metastatic process and detect patients at risk for developing metastatic disease early, in order to intervene earlier and improve their survival [2].

Metastasis is a complex process involving many steps. For example, in order to form a clinically significant metastasis through the hematogenous route, a cancer cell must detach from the cells surrounding it (a process known as the epithelial to mesenchymal transition or EMT), invade the surrounding extracellular matrix, enter and survive in the peripheral circulation, exit the circulation in an organ suitable for metastasis, and then survive and proliferate in the new environment [3]. A cancer cell has to accumulate many genetic and epigenetic alterations in order to acquire all of the above functions necessary to become metastatic. Many of these alterations may manifest themselves though altered gene expression. The interruption of any one or more of these steps could potentially inhibit the development of clinically significant metastasis [3]. More than 32 genes that are important in suppressing the development of metastasis in one or more human cancers have been identified to date [4].

### Rationale

While different cancer types do not necessarily have the same genetic program for metastasis, Ramaswamy *et al *described a single molecular signature of metastasis, identified in the comparison of metastatic and non-metastatic adenocarcinomas, that could predict outcomes in various cancers, including breast, prostate, and even medulloblastoma, a non-epithelial pediatric brain tumor [5]. It was therefore our hypothesis that there are common genes and pathways of metastasis shared by multiple cancer types, and that by expanding the above analysis to incorporate more or diverse tumor types, we would be able to identify more reliable genes and pathways involved in these common steps.

The large number of expression microarray datasets in the public domain provides a rich resource for genome-wide information on cancer and affords an opportunity to perform meta-analysis with a large number of cases. Meta-analysis consists of statistical techniques to combine results from several studies in order to increase statistical power and reproducibility compared with any single study [6]. Rhodes *et al *successfully used meta-analysis to identify a common transcriptional profile that is universally activated in most cancer types relative to the normal tissues from which they arose, likely reflecting essential transcriptional features of neoplastic transformation [7]. Parmigiani *et al *also successfully applied meta-analysis of gene expression to the molecular classification of lung cancer [8].

### Objectives

In this study, we hypothesize that a meta-analysis of publicly available genomic expression datasets of various cancer types can identify a common metastatic signature of metastasis. We tested this hypothesis by implementing and applying a modified permutation meta-analysis method on multiple microarray datasets and then validated the signature in independent datasets.

## Methods

### Eligibility Criteria

We searched the public cancer microarray database, Oncomine [9], to identify expression microarray datasets that compared the expression of primary tumors versus distant metastases of various cancer types. In order to be included in our study, a dataset was required to (1) be generated from human tumors, (2) compare primary tumors versus distant metastatic tumors, (3) have at least one significant gene with a Q-value < 0.1, and (4) not include samples that overlapped with those of another identified dataset. In addition, we eliminated two datasets with > 50% of the tested genes with Q-values < 0.1 because of a potential quality issue with the dataset.

### Information sources

Oncomine is the most comprehensive cancer-specific database currently containing 512 datasets investigating 35 tumor types [9,10]. This database was an excellent source of datasets for this study because the datasets contained additional sample information, which was easily accessible and analyzable. The data obtained is processed by the Oncomine team prior to export. Expression values are log-transformed and median-centered per array. Differential expression is identified by a permutation test with shrinkage to reduce the noise in the data, and false discovery rates (Q-value = NP/R, where P is the p-value, N is the total number of genes analyzed, and R is the sorted rank of P) are calculated to correct for multiple testing [11].

### Search/Study selection

We performed a simple search for the search term "met" and obtained 42 studies of which 5 were quickly eliminated as being non-human studies or for having evaluated late metastases on primary tumors, instead of having a sample from the metastasis. We then identified 37 studies [5,12-43] in the Oncomine database that were analyzed on the basis of primary tumor versus metastasis. Cited literature was reviewed to confirm that the analysis was as documented in the Oncomine database.

### Data collection process/Data items

For each of the identified studies, data for all genes with a Q-value less than 0.1 were extracted from the database as a .csv file. Since Oncomine does not allow the export of the raw data, the extracted data included gene symbol, reporter ID, mean expression levels in the primary and metastatic tumors, p-value, and calculated Q-value of each feature or gene.

### Summary measures

Since the raw data were not available for some of these studies, we decided to use a counting method that could make us of the differential expression information from the Oncomine database for the meta-analysis. We modified a meta-analysis method that was originally reported by Rhodes et al. [7] and implemented it in the R statistical computing environment [44]. This method essentially counts the number of datasets in which each gene is significantly differentially expressed and performs permutations in order to determine the significance of being differentially expressed in each number of studies. Our improvement on the method involved the definition of the False Discovery Proportion (FDP) which was a cumulative measure of the false discovery rate that smoothed the curve as the number of repeated genes decreased and was more sensitive in identifying genes of interest than the original method based on comparative analysis of the two methods. This measure, FDP_k_, is the number of genes present in k or more studies as found by random permutation divided by the observed number of genes present in k or more studies. The code for this implementation is available upon request. The algorithm included the following steps:

▪ A set of *S *differential expression datasets was selected.

▪ For each dataset, two signatures were created. One consisted of unique genes that were under-expressed metastases versus primary tumors in the dataset with a Q-value < 0.1, and the other consisted of those unique genes over-expressed with a Q-value < 0.1.

▪ Each of the following steps of the algorithm was performed separately for the over- and under-expressed genes.

▪ For each gene, the number of signatures (from *0 *to *S*) in which it was present was counted (i.e. the number of studies in which it was significantly differentially expressed between primary and metastases.)

**Figure 1:**
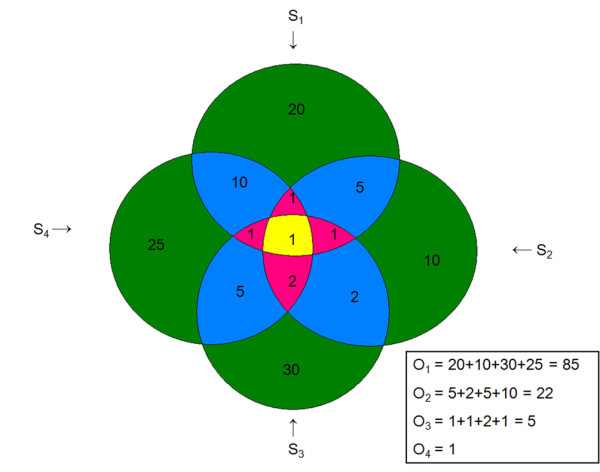
**Example of the identification of the *O_i _*in the meta-analysis method**. Each of the circles represents a hypothetical dataset (*S_1 _*to *S_4_*). The numerals are the number of genes differentially expressed in the datasets represented by that area of overlap of the circles. The value *O_i _*is defined as the number of genes differentially expressed in exactly *i *number of datasets. In the example, *O_1 _*is 85 since that is the number of genes differentially expressed in 1 study, whereas *O_4 _*is 1 since only one gene is present in the area overlapping all 4 studies.

▪ The total number of genes, *O_j_*, present in exactly *j *signatures was tallied (*O_1_, O_2_*, ..., *O_S_*). (For a hypothetical example, see Figure 1.)

▪ Random permutations were performed in which the same set of Q-values was randomly assigned to the unique genes within each study, so that the set of genes in each signature changed at random, but the number of significant genes in each individual study and the genes at risk for inclusion remained the same.

▪ Each permutation generated a tally of the number of genes, *E_j_*, found to be present in j random signatures by chance alone (*E_1_, E_2,_*...,*E_S_*)

▪ The procedure was repeated 1000 times resulting in a matrix *E_ij _*where *i *was the permutation and *j *was the number of signatures

▪ A False Discovery Proportion was calculated for each number of studies *k *where

▪ An FDP < 0.1 was considered significant. The genes that met the FDP cutoff were selected as the common metastatic signature of metastasis.

### Other analyses

#### Pathway analysis

Using the commercial pathways knowledge database Ingenuity Pathways Analysis (IPA), we identified canonical pathways that were enriched or over-represented in the common metastatic signature [45]. Canonical pathway analysis identified the pathways from the IPA library of canonical pathways that were most significant to the metastatic signature. Genes in the signature of metastasis that were associated with a canonical pathway in Ingenuity's Knowledge Base were considered for the analysis. The significance of the association between the signature and the canonical pathway was measured in 3 ways: 1) A ratio of the number of genes from the signature that map to the pathway divided by the total number of genes that map to the canonical pathway was calculated; 2) A right-sided Fisher's exact test was used to calculate a p-value determining the probability that the association between the genes in the dataset and the canonical pathway is explained by chance alone; 3) Benjamini-Hochberg (B-H) method of multiple testing correction was performed [46]. We then performed PubMed literature review to test if the pathways that were significant with a B-H p-value less than 0.05 had been previously implicated in metastasis and mapped these pathways to the known metastatic cascade.

### Validation

In order to perform bioinformatic validation of the gene signature, we downloaded the raw data for the ten studies identified in the Oncomine database that were reserved for validation as described above. After importing and processing the raw data in BRB-ArrayTools v3.7.0 [47], we used the Gene Set Expression Comparison tool to compute the LS statistic p-value for our metastatic signature for each study. The LS statistic tests whether the average degree of differential expression is greater than expected from a random sample of genes. For a set of *N *genes, the LS statistic is defined as

where the *p_i _*are the p-values of the appropriate single gene univariate test. The statistical significance of a gene set, i.e. the LS statistic p-value, is then determined by comparison of the LS statistic to the empirical distribution of LS in random samples of *N *genes. If significant, it provides evidence that the genes within our metastatic signature are differentially expressed between primary tumors and metastases within the validation dataset more often than would be expected by chance alone [48]. This was performed separately for our up-regulated and down-regulated genes. For instance, for the down-regulated genes, the metastatic signature down-regulated genes were analyzed in a filtered list of all the down-regulated genes in the validation dataset.

## Results

### Study Selection

**Figure 2:**
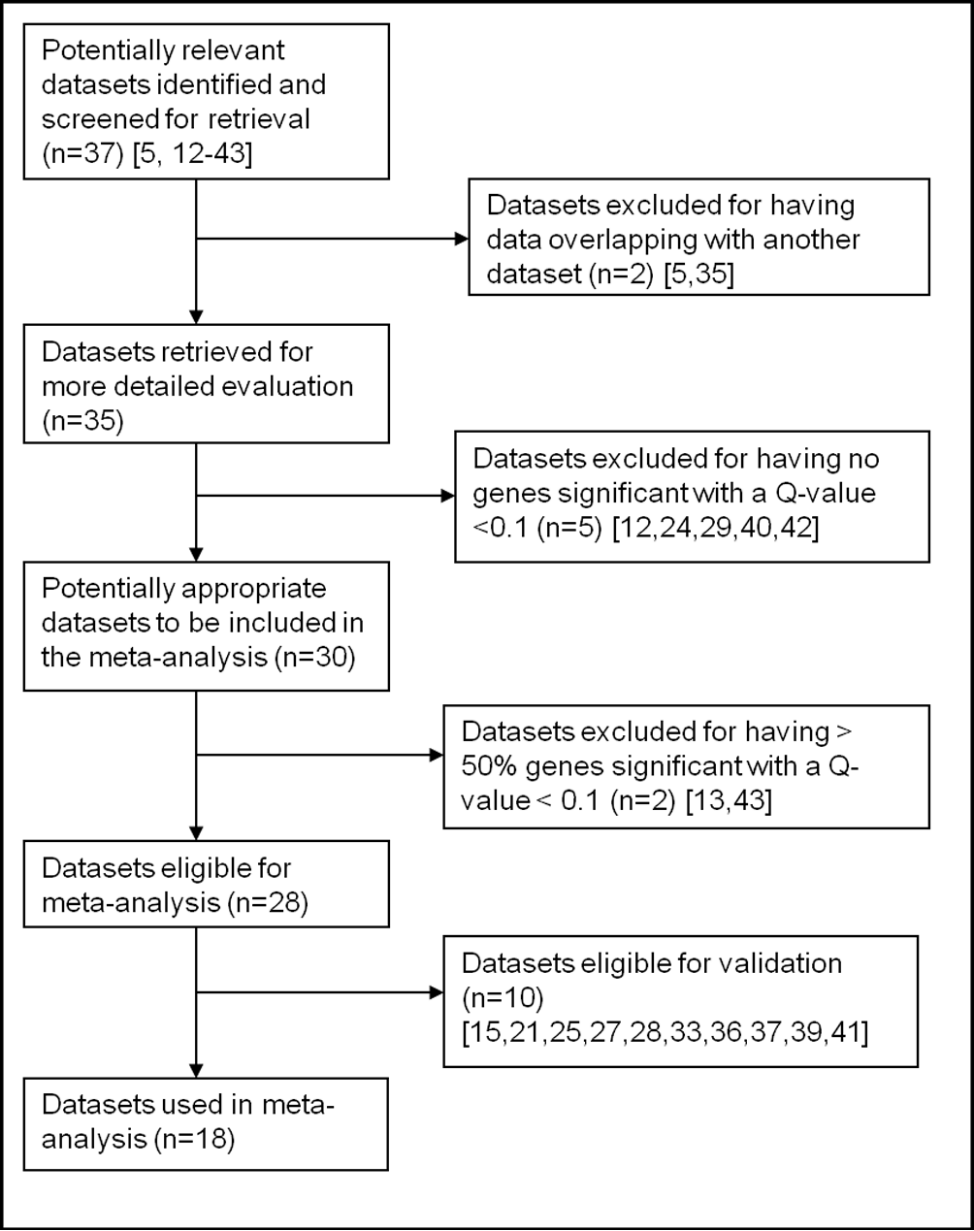
**Flow diagram of the selection of datasets included in the meta-analysis**. After initial screening and identification of potential datasets in the Oncomine database, the process of elimination of ineligible studies is outlined. n: number of datasets in a specific category; numbers in brackets: reference for the dataset.

**Figure 3:**
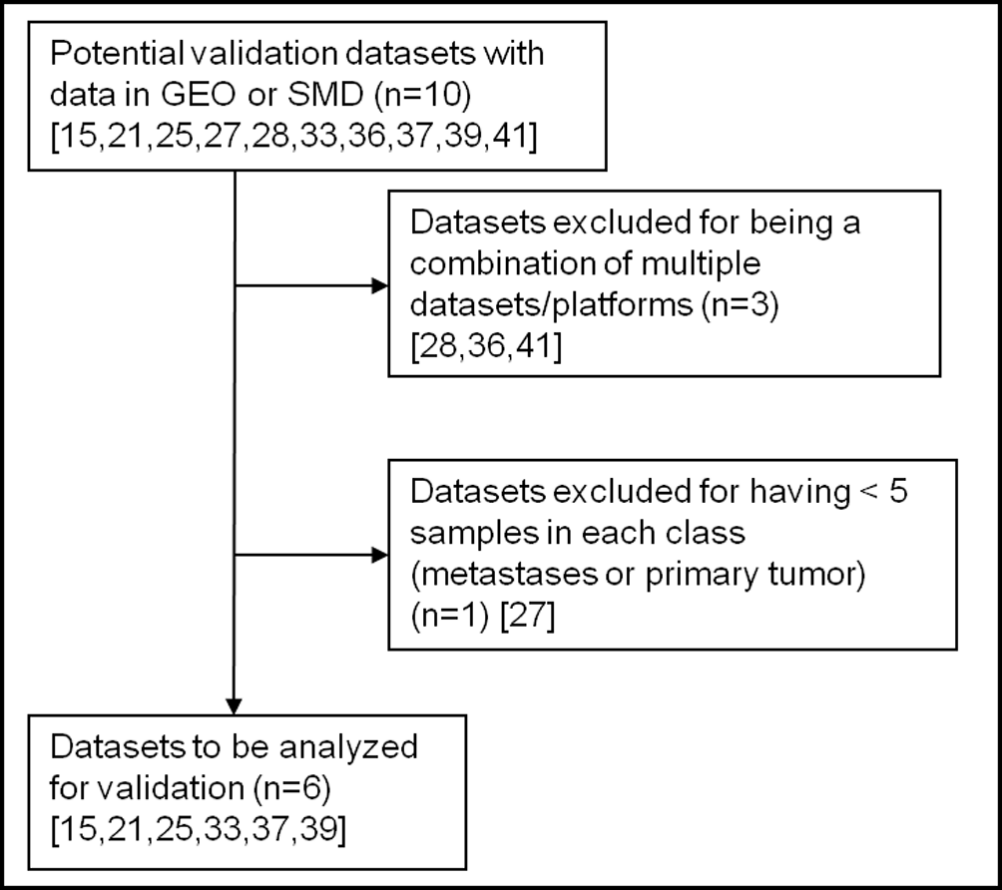
**Flow diagram of the selection of studies included for validation**. The process of selection of possible validation datasets is outlined. n: number of datasets in a specific category; numbers in brackets: reference for the dataset described; GEO: Gene Expression Omnibus [49]; SMD: Stanford Microarray Database [50].

The datasets were obtained from Oncomine, and eligible datasets were selected as outlined in Figure 2. Two were eliminated for potentially having overlapping data with a previously identified dataset [5,35]. Thirty of the remaining datasets (81% of the initial 37 datasets) were found to have significantly altered genes with a Q-value < 0.1. Twenty eight of these (75.6% of the initial 37 datasets) met our eligibility criteria and were included in this analysis. The eligible datasets analyzed the genomic expression of primary tumors versus metastases in multiple different tumor types including melanoma and sarcomas, in addition to various adenocarcinomas. We then set aside ten datasets that had downloadable raw data in the Gene Expression Omnibus (GEO) [49] or the Stanford Microarray Database (SMD) [50] for possible use as validation sets. Upon further review, four validation datasets were eliminated for reasons outlined in Figure 3. The eligible datasets involved various tumor types, including colon cancer, prostate cancer, melanoma, sarcoma, and ovarian cancer.

### Study characteristics

The datasets that were selected for analysis along with their respective number of unique genes tested and the number of genes with a Q-value less than 0.1 are listed in Table 1. The references and accession numbers are also provided in the table.

**Table 1:** Expression microarray studies used in the meta-analysis

	Study [Reference]	Platform	Unique Genes Tested	Genes Sig Up (% of tested)	Genes Sig Down (% of tested)	Primary tumors	Distant Mets	GEO Accession
1	Bittner Breast [14]	HG U133 Plus 2.0	19079	33 (0.2%)	0 (0%)	327	9	GSE2109
2	Bittner Colon [14]	HG U133 Plus 2.0	19079	656 (3.4%)	3938 (20.6%)	330	43	GSE2109
3	Bittner Lung [14]	HG U133 Plus 2.0	19079	127 (0.6%)	15 (0.1%)	101	8	GSE2109
4	Bittner Ovarian [14]	HG U133 Plus 2.0	19079	494 (2.6%)	131 (0.7%)	166	75	GSE2109
5	Bittner Sarcoma [14]	HG U133 Plus 2.0	19079	4 (0%)	1 (0%)	42	10	GSE2109
6	Garber Lung [16]	Institutional cDNA microarray	10723	9 (0.1%)	57 (0.5%)	61	6	GSE3398
7	Graudens Colon [17]	Institutional cDNA microarray	6242	145 (2.3%)	80 (1.3%)	18	30	GSE3964
8	Haqq Melanoma [18]	Research Genetics cDNA microarray	7344	420 (5.7%)	639 (8.7%)	6	19	N/A
9	Holzbeierlein Prostate [19]	HG U95A-Av2	7820	11 (0.1%)	295 (3.8%)	40	9	N/A
10	Jain Endocrine [20]	HG U95A-Av2	7820	14 (0.2%)	229 (2.9%)	8	17	N/A
11	Lapointe Prostate [22]	Institutional cDNA microarray	10021	1081 (10.8%)	1219 (12.2%)	62	9	GSE3933
12	LaTulippe Prostate [23]	HG U95A-Av2	7820	265 (3.4%)	245 (3.1%)	23	9	N/A
13	Magee Prostate [26]	HG FL	4564	35 (0.8%)	18 (0.4%)	8	3	N/A
14	O'Donnell Oral [30]	HG U133A	12427	1 (0%)	28 (0.2%)	22	5	GSE2280
15	Radvanyi Breast [31]	Custom cDNA microarray	16133	548 (3.3%)	85 (0.5%)	47	7	GSE1477
16	Ramaswamy Multicancer [32]	HG FL, Hu35KsubA	9064	556 (3.4%)	301 (3.3%)	10	4	N/A
17	Segal Sarcoma [34]	HG U95A-Av2	7820	168 (2.1%)	164 (2.1%)	29	4	N/A
18	Vanaja Prostate [38]	HG U133A, HG U133B	17358	4 (0%)	208 (1.2%)	27	5	N/A

The 18 datasets used in the meta-analysis are described with regard to the platform used in the original experiment, the number of unique genes represented in the platform, the number of genes significantly (sig) dysregulated in metastases compared with primaries with a Q-value < 0.1, the number of samples that are primary tumors or distant metastases (mets), and the Gene Expression Omnibus (GEO) Accession number. HG U133 Plus 2: Affymetrix Human Genome U133 Plus 2.0 Array; HG U95A0Av2: Affymetrix Human Genome U95A-Av2 Array; HG FL: Affymetrix HumanGeneFL Array; HG U133A: Affymetrix Human Genome U133A Array: HG U133B: Affymetrix Human Genome U133B Array; N/A: Not applicable.

### Synthesis of results

**Figure 4:**
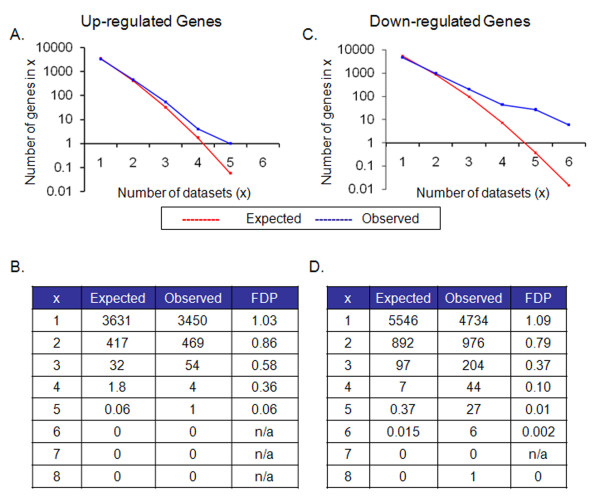
**Average observed by permutation versus observed dysregulated genes found by the meta-analysis method**. The number of genes expected to be repeated as calculated by our permutation method and the number of repeated genes observed in our datasets is plotted against the number of studies (x) in which they are repeated (4*a *shows the results for the up-regulated genes; 4*c *is the results for the down-regulated genes). The actual numbers are presented in the tables below the corresponding chart (4*b *corresponds with up-regulated, and 4*d *with down). The observed repeated genes are greater than the expected number when significant in 2 datasets. This was considered significant when the FDP < 0.1.

**Figure 5:**
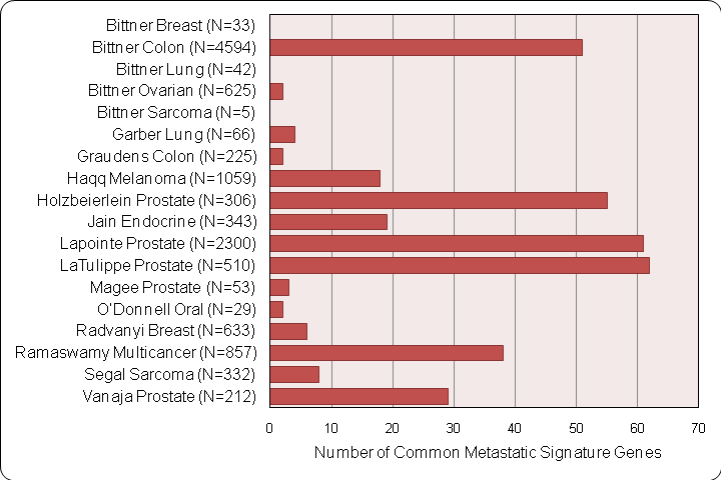
**Number of genes in the common metastatic signature significant (Q-value < 0.1) in each dataset**. N: Number of unique genes differentially expressed with a Q-value < 0.1.

To identify a common metastatic signature in solid tumors, we implemented a modified permutation counting method in the R statistical environment in order to perform a meta-analysis of 18 publicly available expression microarray datasets extracted from the Oncomine database (Table 1). Based on the meta-analysis, we discovered that down-regulated genes that were present in four or more studies and up-regulated genes in five or more studies were more prevalent than would be expected by chance alone with a False Discovery Proportion (FDP) of less than 0.1 (See Figure 4). Interestingly, we identified 78 (44 + 27 + 6 + 1) down-regulated genes and only 1 up-regulated gene in metastases compared with primary tumors. These differentially expressed genes constituted a common signature of metastasis and are listed in Table 2. As expected, those datasets that had more differentially expressed genes contributed more to the metastatic signature than those with fewer genes (See Figure 5). However, all but three of the datasets contributed at least one gene to the common metastatic signature. The specific datasets in which a specific signature gene was significant with a FDP < 0.1 are listed in the Table 3.

**Table 2:** The Common Metastatic Signature

Number of studies repeated	Up-regulated in metastasis	Down-regulated in metastasis			
4 studies	Not significant	ACTG2	GJA1	NBL1	RARRES1
		CASP7	GNG12	PAGE4	SELE
		CSRP1	GSN	PAM	SLC12A4
		CYR61	IER2	PCP4	SMTN
		DPT	ISL1	PDE4D	SORBS1
		DSTN	JMJD3	PIGB	SYNPO2
		FILIP1L	JUNB	PKIG	TCF21
		FLNC	KRT15	PLA2G2A	TMEM49
		FOSB	LUM	PLEKHC1	TPM1
		FUCA1	MAPK1	PPP1R12A	TSC22D1
		GADD45B	MFAP4	RAP1A	VCL
5 studies	EZH2	ACTA2	DKFZP564O0823	LMOD1	RBPMS
		BMPR1A	DMN	MCL1	SPARCL1
		CAMK2G	FBLN1	MGP	SPG20
		CCND2	FHL1	NR4A1	TACC1
		CNN1	FXYD3	NR4A3	TAGLN
		CTGF	HBEGF	PPP1R12B	ZFP36
		DIO2	KCNMA1	PYROXD1	
6 studies	None	BTG2	KCNMB1	MYLK	
		JUND	MYH11	SOD3	
8 studies	None	TPM2			

The gene symbols of the genes in the metastatic signature are given with along with the number of studies in which it was significant.

**Table 3:** Datasets in which genes in common metastatic signature of metastasis are significant with a Q < 0.1

Genes differentially expressed in metastasis	1	2	3	4	5	6	7	8	9	10	11	12	13	14	15	16	17	18	Num studies
**Up - regulated genes**																			
EZH2											X	X	X		X	X			5
**Down - regulated genes**																			
ACTG2		X					X				X							X	4
FUCA1								X		X	X	X							4
JUNB									X		X	X				X			4
PDE4D		X							X		X					X			4
RAP1A		X							X		X						X		4
SYNPO2		X									X					X		X	4
CASP7		X					X				X							X	4
GADD45B									X	X		X				X			4
KRT15											X	X				X		X	4
PIGB								X		X	X							X	4
RARRES1		X							X		X	X							4
TCF21		X		X								X						X	4
CSRP1		X							X			X						X	4
GJA1								X	X	X	X								4
LUM		X				X		X			X								4
PKIG		X							X			X					X		4
SELE									X		X	X						X	4
TMEM49								X			X				X	X			4
CYR61									X	X	X	X							4
GNG12		X							X		X					X			4
MAPK1		X						X			X					X			4
PLA2G2A		X							X		X	X							4
SLC12A4								X				X				X		X	4
TPM1		X							X		X	X							4
DPT		X						X			X						X		4
GSN		X							X		X	X							4
MFAP4		X				X					X	X							4
PLEKHC1									X		X	X				X			4
SMTN		X							X			X					X		4
TSC22D1									X		X	X				X			4
DSTN								X	X		X	X							4
IER2		X							X			X				X			4
NBL1		X							X			X				X			4
PPP1R12A		X				X			X		X								4
SORBS1		X									X					X		X	4
VCL		X							X			X						X	4
FILIP1L		X							X		X	X							4
ISL1									X		X	X						X	4
PAGE4											X	X				X		X	4
FLNC		X									X	X						X	4
JMJD3		X							X			X				X			4
PAM								X	X		X					X			4
FOSB									X	X		X	X						4
PCP4											X	X				X		X	4
ACTA2								X	X		X	X				X			5
CNN1		X						X			X	X						X	5
DMN		X										X		X		X		X	5
HBEGF		X						X	X	X	X								5
MGP									X		X	X				X		X	5
PYRO1D1		X							X		X	X					X		5
TACC1		X				X			X		X					X			5
BMPR1A		X							X		X	X						X	5
CTGF								X	X	X	X	X							5
FBLN1		X						X	X		X	X							5
KCNMA1		X						X				X			X	X			5
NR4A1		X							X			X	X			X			5
RBPMS									X	X	X	X					X		5
TAGLN									X		X	X				X		X	5
CAMK2G		X							X	X		X				X			5
DIO2		X								X	X	X						X	5
FHL1		X							X	X	X	X							5
LMOD1		X									X	X				X		X	5
NR4A3								X	X	X		X				X			5
SPARCL1		X							X		X	X			X				5
ZFP36									X	X	X	X				X			5
CCND2		X							X		X	X				X			5
DKFZP564O0823		X							X		X	X						X	5
FXYD3		X		X						X	X	X							5
MCL1									X		X	X				X	X		5
PPP1R12B		X							X		X	X						X	5
SPG20									X	X	X	X			X				5
BTG2		X							X	X	X	X				X			6
JUND		X						X	X	X		X				X			6
KCNMB1		X							X		X	X				X		X	6
MYH11		X							X		X	X				X		X	6
MYLK		X							X		X	X				X		X	6
SOD3		X							X	X		X					X	X	6
TPM2		X							X		X	X		X	X	X		X	8

The studies in which each gene is differentially expressed are given as an × under the study number, as given in Table 1. "Num studies" refers to the total number of studies in which the gene is significantly differentially expressed with a Q-value < 0.1.

This study expanded upon the previous study by Ramaswamy *et al *by including multiple cancer types, as opposed to only adenocarcinomas [5]. Our method was able to capture 5 of the 17 genes in the Ramaswamy metastatic signature. These genes were found to be down-regulated in both signatures: ACTG2, MYLK, MYH11, CNN1, and NR4A2. The only up-regulated gene, EZH2, identified in our study was not part of the Ramaswamy signature though the gene was up-regulated with respect to metastasis in the Ramaswamy Multi-cancer dataset comparing primary tumors versus metastases in Oncomine with a Q-value of 0.05. This suggests that our meta-analysis procedure may identify additional metastatic genes that have not been reported before, but are supported by multiple expression studies.

### Other analyses

#### Pathway analysis of genes involved in the common metastatic signature

**Figure 6:**
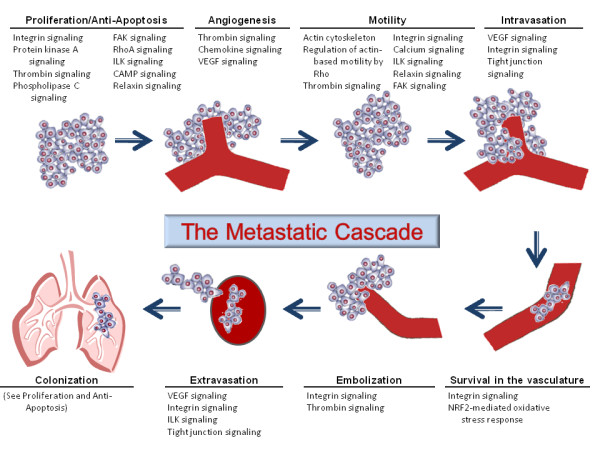
**Mapping of common pathways represented by gene list to metastatic cascade**. Ingenuity pathways significantly enriched by the common metastatic signature with a p-value < 0.01 were mapped to the metastatic cascade after a literature review. The figure is based on the metastatic cascade as published by Isaiah Fidler in 2003 [3].

To validate whether this signature contains metastasis information, we identified the pathways that are significantly enriched in the signature. Ingenuity Pathway Analysis revealed that the down-regulated genes in the metastatic signature were enriched in many pathways previously implicated in metastasis, such as integrin signaling, calcium signaling, and VEGF signaling. The significant pathways are shown in Table 4. In order to determine whether these pathways were potentially specific to the EMT, and therefore epithelial tumors, or whether they could represent common steps that could be shared with non-epithelial tumors, we mapped these pathways to steps in the metastatic cascade in which they had been previously implicated in the literature. Interestingly, each one of the steps in the metastatic cascade has been reported to be associated with one or more of our significant pathways (See Figure 6), suggesting the metastatic signature potentially contains information throughout the whole metastatic cascade.

**Table 4:** Ingenuity canonical pathways significantly (B-H p-value < 0.05) represented by the genes down-regulated in the common metastatic signature

Ingenuity Canonical Pathways	Fisher Exact p-value	B-H p-value	Ratio
Actin Cytoskeleton Signaling	7.94E-08	1.51E-05	4.26E-02
Regulation of Actin-based Motility by Rho	3.89E-06	2.14E-04	6.52E-02
Integrin Signaling	4.57E-06	2.14E-04	3.96E-02
Calcium Signaling	2.24E-05	7.08E-04	3.41E-02
Protein Kinase A Signaling	1.12E-04	3.02E-03	2.51E-02
RhoA Signaling	1.86E-04	4.27E-03	4.55E-02
NRF2-mediated Oxidative Stress Response	2.00E-04	4.27E-03	3.28E-02
ILK Signaling	2.51E-04	4.68E-03	3.23E-02
Thrombin Signaling	3.39E-04	5.75E-03	2.94E-02
Chemokine Signaling	4.17E-04	6.61E-03	5.33E-02
VEGF Signaling	8.32E-04	1.07E-02	4.12E-02
FAK Signaling	8.71E-04	1.07E-02	4.00E-02
Phospholipase C Signaling	9.12E-04	1.07E-02	2.34E-02
cAMP-mediated Signaling	1.02E-03	1.15E-02	3.11E-02
Tight Junction Signaling	1.15E-03	1.20E-02	2.99E-02
Relaxin Signaling	3.47E-03	2.95E-02	2.68E-02
CDK5 Signaling	9.12E-03	6.17E-02	3.19E-02
IL-8 Signaling	9.33E-03	6.17E-02	2.15E-02

B-H: Benjamini-Hochberg method for correcting for the multiple testing problem; Ratio: The number of genes from the metastatic signature that map to the pathway divided by the total number of genes that map to the canonical pathway.

### Validation of the common metastatic signature

To validate that the common metastatic signature could be applied to other metastatic datasets, we performed Gene Set Expression Comparison analysis on six independent gene expression datasets identified through Oncomine that were not used in the meta-analysis. We found that the common metastatic signature was significantly enriched in four out of six of the publicly available datasets (See Table 5). The signature was enriched, with an LS statistic p-value < 0.05, in prostate cancer, gastric cancer, colon cancer, and melanoma datasets [15,21,33,39]. This supports our hypothesis that this common metastatic signature is enriched in multiple tumor types. There were two datasets, an ovarian and sarcoma dataset, tested in which the common metastatic signature was not significantly enriched [25,37]. This may be due to the fact that these tumor types are not well represented in our discovery set and the number of significant genes of these tumor types in the discovery sets is not high. We did attempt to vary the Q-value cutoff from 0.01 to 0.2 in order to vary the number of significant genes represented from these datasets, but this did not improve the validation results. Further studies may need to be performed to test this possibility when more of datasets of these tumor types are available. However, the common metastatic signature was enriched in other tumor types that were underrepresented in our meta-analysis, such as gastric cancer that was not present in the discovery set, suggesting that the validation results are not simply due to the presence or absence of the same tumor types in the discovery and validation sets.

**Table 5:** Enrichment of the common metastatic-signature (CMS)

Study [Reference]	Platform	Unique Genes Tested	Primary Tumors	Distant Mets	Unique CMS Genes in dataset	Significant CMS Genes	LS Statistic p-value	GEO Acc # or SMD Pub #
Varambally Prostate [39]	HG U133 Plus 2.0	19079	7	6	65	57	< 0.00001	GSE3325
Chen Gastric [15]	Undefined cDNA microarray	10568	89	14	61	26	< 0.00001	SMD Pub # 232
Ki Colon [21]	CMRC-GT	9078	52	28	55	25	0.00011	GSE6988
Riker Melanoma [33]	HG U133 Plus 2.0	19079	16	40	71	17	0.011	GSE7553
Linn Sarcoma [25]	Undefined cDNA microarray	14437	47	10	61	5	0.50	SMD Pub # 287
Tothill Ovarian [37]	HG U133 Plus 2.0	19079	189	54	65	7	0.93	GSE9899

The 6 validation datasets with regard to the platform used in the original experiment, the number of unique genes represented in the platform, the number of samples that are primary tumors or distant metastases (mets), the number of genes in the common metastatic signature (CMS) represented in the platform, the number of CMS genes that were significant with a Q-value < 0.1, the LS statistic p-value, and the Gene Expression Omnibus Accession number (GEO Acc #) or SMD Publication number (SMD Pub #). HG U133 Plus 2: Affymetrix Human Genome U133 Plus 2.0 Array; CMRC-GT: Cancer Metastasis Research Center-Genomic Tree array, Yonsei Cancer Center, Seoul, Korea.

## Discussion

### Summary of the evidence

We have used a meta-analysis method to identify genes are shared and important in metastasis. The fact that these genes are involved in pathways that have been previously implicated in metastasis supports their involvement in the metastatic process. These genes may be useful as potential therapeutic targets or predicting clinical outcome. Since these genes and pathways are common to multiple tumor types as shown by the fact that they are enriched in various tumors, these may be targets that can be exploited in many different tumors. Drug discovery could be performed by finding inhibitors of identified pathways, such as FAK inhibitors.

One promising in silico approach of drug discovery is the use of the Connectivity Map to find drugs that can reverse a gene signature, such as the common metastatic signature, and as a result potentially reverse the metastatic phenotype [51,52]. On preliminary analysis of the metastatic signature by the Connectivity Map, the top molecule that could reverse the common metastatic signature by the permutation analysis was camptothecin. Camptothecin is a topoisomerase I inhibitor that has been shown to induce apoptosis in tumor cells. Irinotecan and topotecan, which are analogs of camptothecin, are currently being used to treat several cancers including colon cancer, ovarian cancer, and gliomas [53]. Interestingly, when ranking the drugs by Anatomical Therapeutic Chemical (ATC) codes, the top three ranking codes were all groups of antipsychotics, which may be related to the ability of some of these compounds to induce autophagy in experimental models [54]. The molecules in these groups were significantly associated with reversal of the metastatic signature with good specificity for the signature. This may represent known and readily available drugs that could have a new application immediately without the need of developing a new compound, which would take many years of testing. The result of this in silico analysis can only be confirmed with more analyses and experiments; however, this shows the promise of applying this signature to the prediction and therapy of metastatic cancers for improving the outcomes of patients.

Though all of the genes in our signature were differentially expressed in more studies than would be expected by chance alone, it is important to note that none of the genes in the common metastatic signature were present in more than 8 of our 18 datasets. This could be caused by many factors, such as heterogeneity of metastatic tumors, dataset quality, and the use of different platforms without uniform representation of genes of interest. This may explain the difficulty many individuals have found in identifying overlapping genes in multiple datasets examining metastasis [55]. In addition, more overlapping genes may not have been identified because of a potential lack of power caused by using a stringent Q-value of 0.1. However, this highlights the usefulness of the meta-analysis approach in identifying significant metastatic genes that repeat more than expected by chance that may not be identified when initially comparing datasets.

In our analysis, we have also noted that the number of down-regulated genes is much greater than the number of up-regulated genes. This intriguing observation suggests that overcoming metastatic suppression may be a critical or common step in tumor progression. Alternatively, the genes involved in metastasis suppression may be more similar and shared among the solid tumors than those involved in metastasis activation processes. It has been previously shown that down-regulation of certain genes, such as KISS1, RhoGDI2, and nm23-H1, is important in metastasis [56]. At least one of our identified down-regulated genes, CDGF, is a recognized metastasis suppressor gene [4]. In addition, two of the most highly dysregulated pathways, the actin cytoskeleton signaling pathway and the regulation of actin motility by Rho, have been associated with multiple metastasis suppressor genes [56]. We expect that further functional studies of the down-regulated genes in our common signature will reveal novel metastasis suppressor genes.

The future applications of this meta-analysis method are numerous, as the number of gene expression datasets increases. For instance, in the field of metastasis, this method could be used to compare patients with primary tumors that are metastatic versus non-metastatic. This may add to the information we have learned from the present study. At the time when study was started, the Oncomine database did not provide enough detailed clinical information to perform this analysis. However, this may be feasible in the future.

### Limitations

As with any meta-analysis, the results are dependent upon the reliability of the original data [6]. However, it was difficult to test the validity of the original experiments without raw data. This quality issue was partially overcome by the use of our criteria to select the studies for the meta-analysis and by the use of the meta-analysis approach itself. Our selection criteria excluded the outliers in our analysis, such as datasets without any significant genes with a Q-value less than 0.1 and those with greater than 50% of the tested genes being significant which we hypothesize might be due to systemic bias rather than true differences. Additionally, the process of combining different studies into one analysis should theoretically minimize the effect of some of the possible confounders or quality issues that may be present in certain studies. Since any gene in our signature had to be repeated in multiple studies (i.e. in four or more for the down-regulated genes), no one study alone could completely invalidate our gene list. Clinical meta-analyses often test for heterogeneity of studies, but this approach has not been extended to the meta-analysis of genomic studies.

There are several areas for improvement that could be addressed in future studies. One limitation we had to overcome was the fact that it was not possible to download the complete datasets from the Oncomine database, and we were unable to find raw data for most of older studies included in our signature. Therefore, we were unable to compare the complete lists of genes tested and potentially capture genes that were only represented in a small number of platforms by performing more advanced meta-analysis methods, specifically one that could provide weighting based on the number of genes and samples in the initial experiment, such as the weighted z-method [57]. Limiting the study to only those datasets with available raw data would have substantially reduced the number of studies and possibly the power to detect genes of interest. The counting method we performed in this study was only dependent on information of the significant gene lists which allowed the use of the maximum number of array studies. In the future, as more datasets are readily available for download, this problem may be overcome. This current limitation, however, does not affect the conclusion that those genes identified in this study are likely to be of importance. We conclude that this method has good specificity but may have less sensitivity (a higher false negative rate) than other meta-analysis approaches.

Inconsistent gene ontology also complicated the analysis in this study. Since the Oncomine database provides only the Gene Symbol and only one other gene identifier that could not be matched for every dataset, the only common identifier between our validation datasets and the datasets used in the meta-analysis was the gene symbol. However, a gene symbol may map to multiple probes, so we could be counting results of different probes in each dataset. We did ensure that we counted each unique gene symbol only once in each direction by manually removing duplicates in our extracted data prior to running the meta-analysis. In addition, the use of gene symbols forced us to eliminate from our comparison many ESTs that could have been found in multiple studies. This highlights the need for a common identifier standardized across platforms, such as Entrez gene IDs, which will help to identify more common metastatic genes.

Lastly, the majority of studies used in the meta-analysis were from epithelial tumors reflecting their predominance in the population and, hence, the microarray studies. Attempts to remove these epithelial cancer datasets, such as prostate cancers, resulted in lack of power to identify significant metastatic genes. This could be due to a more dramatic biological effect of metastasis in these epithelial tumors, or the power of the studies themselves, such as a larger sample size and less tissue heterogeneity, etc. This is a limitation of our study due to the availability of eligible datasets in the Oncomine database. With the accumulation of more datasets for non-epithelial, non-adenocarcinoma tumors, future studies may be able to incorporate them and identify a more refined common signature of metastasis that is applicable to even more tumor types.

## Conclusions

We have developed a modified meta-analysis counting method and applied it to the comparison of primary tumors versus metastases in various tumor types. We identified a list of 78 down-regulated genes and 1 up-regulated gene in metastases compared to primary tumors with a False Discovery Proportion of less than 0.1. Many of these genes are involved in pathways associated with metastasis. After comparing the list of genes generated by our analysis with six independent datasets testing primaries versus metastases, we found that four of the datasets demonstrated that these genes were dysregulated than would be expected by chance alone (i.e. LS-statistic p-value < 0.05). We believe that the identification of this common metastatic signature could facilitate further research in metastasis, such as outcome prediction, drug discovery, and other functional studies.

We have followed the relevant components of the PRISMA 2009 guidelines in the preparation of this manuscript [58].

## Funding

This work was supported by the NIH Training Grant T32 CA115303 (MHD), NCI grants R33 CA97874-05 (SGH) and U01 CA114757-04 (CCL), the Gillson Longenbaugh Foundation (CCL) and Cancer Prevention and Research Institute of Texas (TKM).

## Competing interests

The authors declare that they have no competing interests.

## Authors' contributions

MHD performed the dataset identification and retrieval, programmed the method in the R environment, performed pathway analysis and validation, and drafted the manuscript. SGH helped refine the meta-analysis method and advised on data display. CCL aided in the study design, data analysis, and in the interpretation of results. TKM conceived of, supervised, designed and coordinated the study, and revised the manuscript. All authors read and approved the final manuscript.

## Pre-publication history

The pre-publication history for this paper can be accessed here:

http://www.biomedcentral.com/1755-8794/4/56/prepub
